# Long‐term use of Minimed™ 780G in children and adolescents with type 1 diabetes under real‐world conditions: The benefits of optimal settings

**DOI:** 10.1111/dom.16226

**Published:** 2025-01-31

**Authors:** Bruno Bombaci, Stefano Passanisi, Marco Calderone, Fabio Macrì, Fortunato Lombardo, Giuseppina Salzano

**Affiliations:** ^1^ Department of Human Pathology in Adult and Developmental Age “Gaetano Barresi” University of Messina Messina Italy

**Keywords:** automated insulin delivery, effectiveness, paediatrics, time in tight range

## 1 BACKGROUND

The MiniMed 780G™ (Medtronic, Northridge, CA), launched in 2020, is currently available in more than 40 countries worldwide. This device uses a proportional–integral–derivative algorithm combined with fuzzy logic features, adjusting basal insulin delivery rate based on customizable glycaemic targets (100, 110 or 120 mg/dL) and delivering automated correction boluses. Furthermore, the algorithm features meal detection and safe bolus technologies to minimize post‐meal glycaemic excursions.^1^

Although numerous experimental and real‐world studies have demonstrated the benefits related to the MiniMed 780G™ use in youth with type 1 diabetes (T1D), there is still no strong evidence of its sustained effectiveness beyond 1 year of use.^2^, ^3^, ^4^, ^5^

The aim of this study is to evaluate glycaemic outcomes in a population of children and adolescents with T1D during their first 24 months of Minimed 780G™ system use in real‐world conditions.

## 2 METHODS

A single‐centre, longitudinal, real‐world design was adopted. Children and adolescents with T1D who began using the Minimed™ 780G system between November 2020 and May 2024 were enrolled. Local ethical committee approval was obtained (n. 39‐23). Participants were included if they met the following criteria: a diagnosis of T1D, age <18 years at the time of starting automated insulin delivery (AID) therapy, and provided informed consent for remote access to clinical data.

The following time points were considered across the study period: 2 weeks preceding automatic mode activation (T0), 2 weeks after activation of the automatic mode (T1), 12 months after automatic mode activation (T2), 24 months post‐activation (T3). Prior to May 2022, all participants used a 3‐day infusion set, whereas afterward, all users were gradually switched to a 7‐day infusion set.

At each time point, we collected CGM metrics including mean sensor glucose and its standard deviation (SD), percentage of time between 70 and 180 mg/dL (TIR), percentage of time between 70 and 140 mg/dL (TITR), percentage of time above 180 mg/dL (TAR), percentage of time between 180 and 250 mg/dL (TAR_Level1_), percentage of time above 250 mg/dL (TAR_Level2_), percentage of time below 70 mg/dL (TBR), percentage of time between 54 and 70 mg/dL (TBR_Level1_), percentage of time below 54 mg/dL (TBR_Level2_), glucose management indicator (GMI), coefficient of variation (CV) and glycaemia risk index (GRI). All metrics were acquired from CareLink System® platform, considering time intervals of 15 days.

Numerical data were expressed as mean and standard deviation, while categorical variables were reported as absolute frequencies and percentages. A parametric approach was adopted since the numerical variables followed a normal distribution, as confirmed by the Kolmogorov–Smirnov test. To compare clinical variables, including glycaemic control indicators, across the different time points, paired‐samples Student *t* test was used to compare baseline and the first 2 weeks of automatic mode use, and repeated‐measures analysis of variance (ANOVA) was applied to compare T1, T2 and T3. Post hoc pairwise comparisons were conducted using Tukey test, with Bonferroni correction applied for multiple comparisons, yielding a corrected significance level of 0.017.

At time points T1, T2 and T3, clinical variables and system settings were compared between achievers and non‐achievers of CGM targets of TITR ≥50% and TBR ≤4% using Student *t* test for numerical variables and the chi‐square test for categorical variables. Additionally, a logistic regression model was applied to identify baseline predictors for the simultaneous achievement of TITR ≥50% and TBR ≤4%. Statistical analyses were performed using IBM SPSS software for Windows, Version 22 (IBM Corp., Armonk, NY, United States), with a *p* value of less than 0.05 considered statistically significant.

## 3 RESULTS

We included 111 children and adolescents starting therapy with the Minimed™ 780G, with a slight prevalence of males (55.0%). The mean age of the study participants was 12.7 ± 3.3 years, with an average duration of diabetes of 4.7 ± 3.4 years. Prior to using the Minimed™ 780G system, 46 individuals (41.4%) were on multiple daily insulin injections, while 65 (58.6%) were already using an insulin pump. The average HbA1c in the year preceding enrollment was 7.2% ± 0.9%. At T0, the mean BMI *Z*‐score was 0.8 ± 1.2.

Based on the time of initiation of device use, all participants completed a 6‐month observational period, 97 (87.4%) achieved 12‐month use of the Minimed 780G, and 46 (41.4%) completed the 24‐month follow‐up period.

Immediately after the switch from manual to automatic mode, we observed significant improvement of TBR_Level1_ (2.2 ± 1.7 vs. 1.9% ± 1.7%; *p* = 0.015), TBR (2.6 ± 2.2 vs. 2.4% ± 2.4%; *p* = 0.045), TITR (43.1% ± 10.1% vs. 51.5% ± 8.6%; *p* <0.001), TIR (66.9 ± 10.8 vs. 76.0% ± 7.6%; *p* <0.001), TAR_Level1_ (24.0 ± 7.2 vs. 18.3 ± 5.8; *p* <0.001), TAR_Level2_ (6.5 ± 4.8 vs. 3.6% ± 3.0%; *p* <0.001), TAR (30.6 ± 10.8 vs. 21.9% ± 7.8%; *p* <0.001), CV (34.6 ± 4.0 vs. 33.0% ± 4.0%; *p* <0.001), mean sensor glucose (156.5 ± 15.6 vs. 144.7 ± 11.3 mg/dL; *p* <0.001) and GRI (36.3 ± 12.5 vs. 23.4 ± 8.0; *p* <0.001).

**FIGURE 1:**
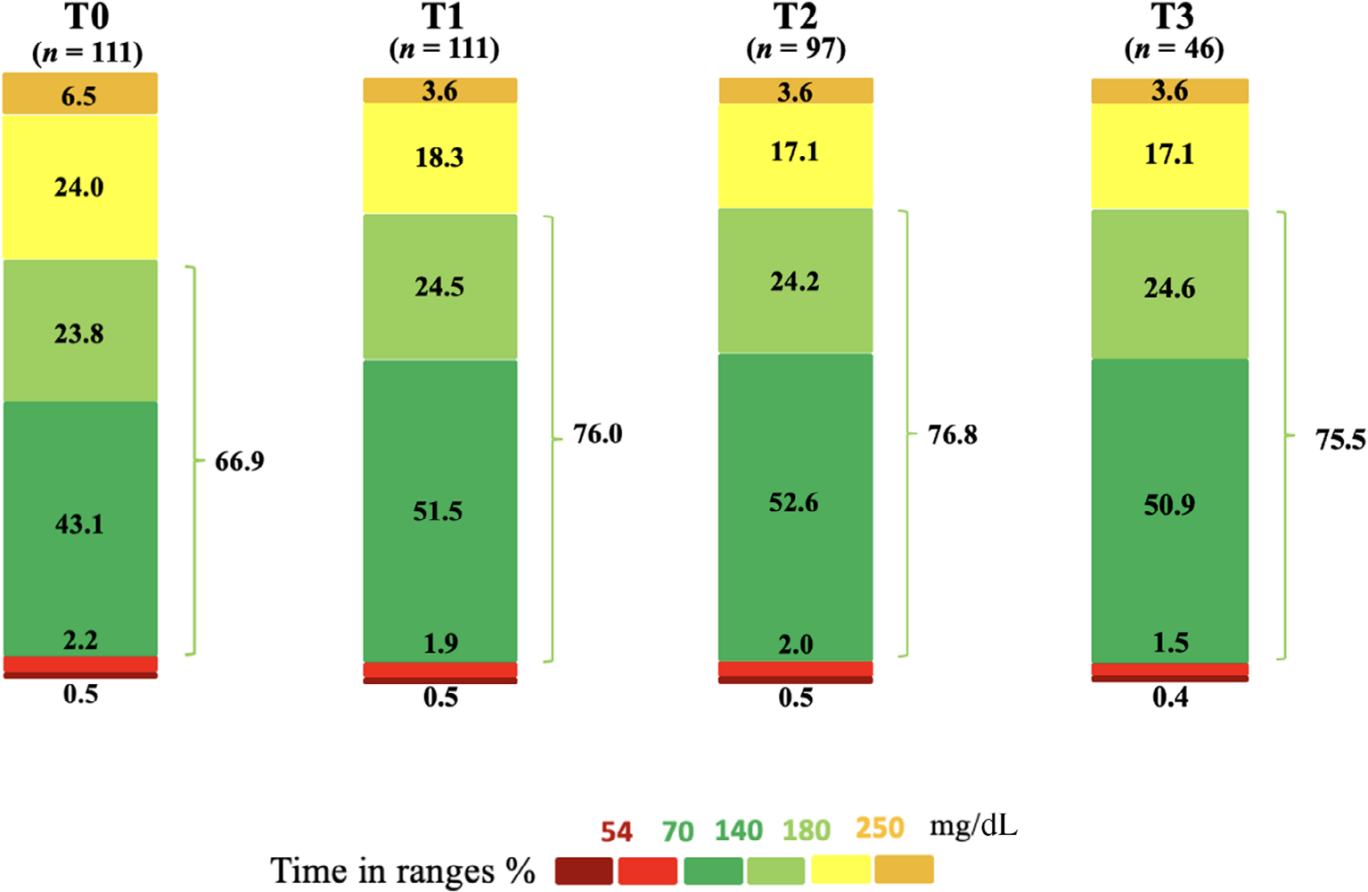
Key CGM metrics of study participants at T0, T1, T2 and T3.

Glucose metrics remained statistically unchanged over the subsequent 24 months of follow‐up, with no significant differences between T1, T2 and T3 (Figure 1).

When comparing achievers and non‐achievers of TITR ≥50% and a TBR ≤4% at T1, we found lower percentage of automatic correction boluses (*p* = 0.009) and more frequent use of active insulin time (AIT) 2 h (*p* = 0.005) and optimal settings, consisting of both AIT 2 h and SmartGuard target 100 mg/dL (*p* = 0.003) among achievers. Similar findings were observed at T2 (Table 1).

**TABLE 1:** Comparison between achievers and non‐achievers of time in tight range ≥50% and a time below range ≤4%.

	T1 target (*n* = 55)	T1 no target (*n* = 56)	*p* value	T2 target (*n* = 52)	T2 no target (*n* = 45)	*p* value	T3 target (*n* = 23)	T3 no target (*n* = 23)	*p* value
Age (years)	13.0 ± 3.3	12.4 ± 3.4	0.293	13.0 ± 3.3	12.4 ± 3.4	0.293	13.0 ± 3.3	12.4 ± 3.4	0.293
SmartGuard use (%)	95.7 ± 8.2	96.7 ± 5.6	0.748	94.8 ± 8.5	95.0 ± 8.0	0.944	97.7 ± 2.3	95.8 ± 6.0	0.992
TDD (IU/kg/day)	0.8 ± 0.2	0.8 ± 0.3	0.509	0.9 ± 0.2	0.9 ± 0.2	0.857	0.9 ± 0.2	0.9 ± 0.2	0.727
Basal (%)	45.4 ± 7.9	43.4 ± 7.4	0.167	41.7 ± 6.8	41.3 ± 6.9	0.589	39.7 ± 6.8	40.2 ± 5.4	0.741
Automated corrections boluses (%)	29.6 ± 8.0	33.9 ± 9.9	0.009*	30.0 ± 8.9	38.4 ± 11.6	<0.001*	30.9 ± 9.4	41.0 ± 12.3	0.005*
Boluses (%)	54.6 ± 7.9	56.6 ± 7.4	0.337	58.3 ± 6.9	41.3 ± 6.9	0.748	60.3 ± 6.8	59.8 ± 5.4	0.303
ICR/day	1.9 ± 1.1	1.8 ± 0.8	0.714	2.7 ± 1.0	2.7 ± 1.0	0.857	3.0 ± 1.0	3.0 ± 1.1	0.984
CHO input/day	4.0 ± 1.7	4.7 ± 2.0	0.030	3.9 ± 1.4	3.9 ± 1.8	0.849	3.8 ± 1.6	3.3 ± 1.5	0.218
SmartGuard target 100 mg/dL (%)	83.6	67.9	0.052	78.8	71.1	0.387	74	78.3	0.729
AIT 2 h (%)	65.5	39.3	0.005*	86.5	66.7	0.019*	74	82.6	0.720
Optimal settings (%)	56.4	28.6	0.003*	73.1	51.1	0.025*	60.9	69.6	0.535

Abbreviations: AIT, active insulin time; CHO, carbohydrate; ICR, insulin/carbohydrate ratio; TDD, total daily dose.

* Significant *p* value <0.05.

The logistic regression analysis did not identify any baseline predictors for the simultaneous achievement of TITR ≥50% and TBR ≤4%, except for an inverse association between the mean HbA1c value in the 12 months prior to enrollment and target achievement at T2 (*B* = −1.126; 95% CI 0.173–0.607; *p* <0.001).

## 4 CONCLUSIONS

Our data confirm the immediate benefits of the Minimed™ 780G use in automatic mode, resulting in significant improvements of key CGM metrics, including TIR and TITR, and reduction in hypoglycaemic events. These results align with previous studies that reported substantial short‐term benefits of AID systems in youths, starting within 2 weeks after the activation of automatic mode.^5^, ^6^

Notably, our data revealed that improvements in CGM data were sustained up to 24 months, with no significant differences between 12‐ and 24‐month metrics. Similarly, a prospective study on 50 youths showed sustained performance of the device in terms of TIR across 2‐year study period.^7^ Another follow‐up analysis on 35 preschoolers participating to a non‐randomized, prospective, single‐arm clinical trial, revealed persistence of positive effect of the Minimed™ 780G on glycaemic control up to 18 months.^8^ The stability of glycaemic outcomes up to 2 years is particularly noteworthy given the real‐world challenges of device adherence and behavioural variability in children and adolescents. Diabetes management in youths is often characterized by numerous challenges, including specific physiological factors that bring significant daily glucose excursions and psychological and behavioural issues typical of this age, with a significant portion of adolescents not achieving recommended glycaemic targets.^9^, ^10^

In our analysis, we focused on the characteristics of subjects achieving a TITR of 50% without exceeding the recommended threshold for time spent in hypoglycemia. TITR has been recently proposed as an alternative metric to TIR for clinical practice, as it more accurately reflects time spent in the euglycaemic range. Interestingly, we found that participants who met glycaemic targets of TITR ≥50% and TBR ≤4% were more likely to use more stringent device settings. However, this association was not confirmed after 24 months. Further studies with larger cohorts are needed to validate this trend and to elucidate potential underlying determinants. AIT of 2 h along with SmartGuard target set at 100 mg/dL have already been identified as predictors of improved TIR among 12 870 Minimed™ 780G users.^11^ A subanalysis focusing on individuals aged ≤15 years from the same population further demonstrated that the percentage of individuals simultaneously achieving the recommended targets for TIR, TBR and GMI was significantly higher among those using optimal settings than the general population.^12^ Similarly, consistent use of AIT of 2 h and a SmartGuard target of 100 mg/dL has been strongly associated to higher TITR levels.^6^

Finally, our analysis revealed a reduced percentage of automatic correction boluses among participants who simultaneously achieved TITR and TBR targets. This finding was consistent across all time points and aligns with previous studies demonstrating an association between a higher proportion of automatic correction boluses and suboptimal glycaemic outcomes among AID users.^5^, ^11^ A study on youths with T1D using the MiniMed™ 780G reported that an automatic correction bolus percentage below 30% was associated with higher levels of TIR and TITR.^13^ This trend underscores the critical role of user‐initiated boluses and accurate carbohydrate counting in achieving optimal glycaemic control.^14^

Despite its strengths, our study has some limitations, including the decreasing sample size over the 24‐month period, which reflects the fact that some participants were enrolled more recently and the single‐center design, that may limit the generalizability of the findings.

In conclusion, our study demonstrated that the Minimed™ 780G provides sustained improvements in glycaemic control for up to 24 months in children and adolescents with T1D. Achieving time in tight range targets without increasing the risk of hypoglycemia is strongly associated with the use of optimal device settings.

## AUTHOR CONTRIBUTIONS

BB and SP conceptualized the study and wrote the first draft of the article. FM and MC researched data. GS reviewed and edited the manuscript. FL contributed to discussion and reviewed and edited the manuscript. All authors approved the final version of the manuscript. SP is the guarantor of this work and, as such, had full access to all the data in the study and takes responsibility for the integrity of the data and the accuracy of the data analysis.

## FUNDING INFORMATION

No funding was received for this article.

## CONFLICT OF INTEREST STATEMENT

BB reports travel grants from Movi SpA and Abbott. FL has received speaker and consultant honoraria from Sanofi and speaking honoraria from Movi SpA. SP received speaking honoraria from Movi SpA.

### PEER REVIEW

The peer review history for this article is available at https://www.webofscience.com/api/gateway/wos/peer‐review/10.1111/dom.16226.

## Data Availability

The data that support the findings of this study are available from the corresponding author upon reasonable request.

## References

[1] Passanisi S , Lombardo F , Mameli C , et al. Safety, metabolic and psychological outcomes of medtronic MiniMed 780G™ in children, adolescents and young adults: a systematic review. Diabetes Ther. 2024;15(2):343‐365. 38038896 10.1007/s13300-023-01501-6 PMC10838896

[2] Silva JD , Lepore G , Battelino T , et al. Real‐world performance of the MiniMed™ 780G system: first report of outcomes from 4120 users. Diabetes Technol Ther. 2022;24(2):113‐119. 34524003 10.1089/dia.2021.0203 PMC8817690

[3] Pulkkinen MA , Varimo TJ , Hakonen ET , et al. MiniMed 780G™ in 2‐ to 6‐year‐old children: safety and clinical outcomes after the first 12 weeks. Diabetes Technol Ther. 2023;25(2):100‐107. 36511831 10.1089/dia.2022.0313

[4] Bergenstal RM , Nimri R , Beck RW , et al. A comparison of two hybrid closed‐loop systems in adolescents and young adults with type 1 diabetes (FLAIR): a multicentre, randomised, crossover trial. Lancet. 2021;397(10270):208‐219. 33453783 10.1016/S0140-6736(20)32514-9 PMC9194961

[5] Passanisi S , Salzano G , Bombaci B , et al. Sustained effectiveness of an advanced hybrid closed‐loop system in a cohort of children and adolescents with type 1 diabetes: a 1‐year real‐world study. Diabetes Care. 2024;47(6):1084‐1091. 38626260 10.2337/dc23-2311

[6] Castañeda J , Arrieta A , van den Heuvel T , Battelino T , Cohen O . Time in tight glucose range in type 1 diabetes: predictive factors and achievable targets in real‐world users of the MiniMed 780G system. Diabetes Care. 2024;47(5):790‐797. 38113453 10.2337/dc23-1581 PMC11043222

[7] Seget S , Chobot A , Tarasiewicz M , et al. Glycemic control in children with type 1 diabetes treated with the advanced hybrid closed loop system 2‐year prospective, observational, two‐center study. Front Endocrinol. 2024;15:1332418. 10.3389/fendo.2024.1332418 PMC10882083 38390211

[8] Pulkkinen MA , Varimo TJ , Hakonen ET , Hero MT , Miettinen PJ , Tuomaala AK . During an 18‐month course of automated insulin delivery treatment, children aged 2 to 6 years achieve and maintain a higher time in tight range. Diabetes Obes Metab. 2024;26(6):2431‐2438. 38514384 10.1111/dom.15562

[9] Bombaci B , Torre A , Longo A , et al. Psychological and clinical challenges in the management of type 1 diabetes during adolescence: a narrative review. Children. 2024;11(9):1085. 39334618 10.3390/children11091085 PMC11430186

[10] Battelino T , Danne T , Bergenstal RM , et al. Clinical targets for continuous glucose monitoring data interpretation: recommendations from the international consensus on time in range. Diabetes Care. 2019;42(8):1593‐1603. 31177185 10.2337/dci19-0028 PMC6973648

[11] Castañeda J , Mathieu C , Aanstoot HJ , et al. Predictors of time in target glucose range in real‐world users of the MiniMed 780G system. Diabetes Obes Metab. 2022;24(11):2212‐2221. 35791621 10.1111/dom.14807

[12] Arrieta A , Battelino T , Scaramuzza AE , et al. Comparison of MiniMed 780G system performance in users aged younger and older than 15 years: evidence from 12 870 real‐world users. Diabetes Obes Metab. 2022;24(7):1370‐1379. 35403792 10.1111/dom.14714 PMC9545031

[13] Eviz E , Killi NE , Karakus KE , et al. Assessing the feasibility of time in tight range (TITR) targets with advanced hybrid closed loop (AHCL) use in children and adolescents: a single‐centre real‐world study. Diabet Med. 2024;41(8):e15333. 38671595 10.1111/dme.15333

[14] Petrovski G , Campbell J , Pasha M , et al. Simplified meal announcement versus precise carbohydrate counting in adolescents with type 1 diabetes using the MiniMed 780G advanced hybrid closed loop system: a randomized controlled trial comparing glucose control. Diabetes Care. 2023;46(3):544‐550. 36598841 10.2337/dc22-1692 PMC10148675

